# Special Issue: Advances in SARS-CoV-2 Infection

**DOI:** 10.3390/microorganisms11041048

**Published:** 2023-04-17

**Authors:** Carlo Contini, John Charles Rotondo, Benedetta Perna, Matteo Guarino, Roberto De Giorgio

**Affiliations:** 1 Infectious and Dermatology Diseases, St. Anna University-Hospital of Ferrara, University of Ferrara, 44124 Ferrara, Italy; 2 Department of Medical Sciences, University of Ferrara, 44121 Ferrara, Italy; 3 Center for Studies on Gender Medicine, Department of Medical Sciences, University of Ferrara, 44121 Ferrara, Italy; 4 Department of Translational Medicine, St. Anna University-Hospital of Ferrara, University of Ferrara, 44121 Ferrara, Italy

Coronavirus Disease 2019 (COVID-19) is a life-threatening disease caused by the Severe Acute Respiratory Syndrome Coronavirus 2 (SARS-CoV-2) virus which was first reported in late 2019 in China, from where it then spread worldwide [1]. According to the latest data from the World Health Organization (WHO), since the start of the COVID-19 pandemic, there have been 761,402,282 confirmed cases worldwide and 6,887,000 deaths reported so far as a result of pneumonia complicated by SARS-CoV-2 [2].

Since the beginning of the pandemic, and particularly during the first and second waves, the goal of this Special Issue has been to highlight the crucial aspects of SARS-CoV-2 and the disease in light of the new knowledge that was emerging. A total of 15 manuscripts have been published in this Special Issue. These papers provided insights into epidemiology, pathogenesis, epigenetics [3,4] COVID-19 emergencies in hospital settings [5,6], advanced diagnosis [6,7,8], vaccination [9,10], and SARS-CoV-2 infection in the experimental setting [11]. The high scientific rigor, originality, and, for some of them, the high number of citations obtained, are well evident.

A very interesting aspect that emerged from one of these concerns in particular was the role of certain intracellular bacteria, such as *Chlamydia pneumoniae* and *Mycoplasma pneumoniae*, in influencing the outcome and prognosis of COVID-19-positive patients from both a clinical (respiratory) and instrumental (radiological) point of view, although the influence on the mortality rate was not significant compared to the control group [12]. Such co-infections have also been shown to cause an increase in D-dimer and fibrinogen values; this increases the risk of thrombosis due to their known ability to evoke hypercoagulability [13,14]. Another original study, both pathogenetic and clinical, aimed to test whether testosterone levels were associated with glial fibrillary acid protein (GFAP) and ubiquitin carboxy-terminal hydrolase L1 (UCH-L1), biomarkers of brain injury, in patients with a severe form of COVID-19; the study showed that the traumatic brain injury biomarker UCH-L1 may be associated with the neurological damage observed in severe COVID-19 cases [7]. Furthermore, a negative correlation between UCH-L1 and serum testosterone concentrations implies that testosterone may play a role in the development of neurological sequelae in severely ill COVID-19 patients.

A fair number of manuscripts concerning the diagnosis of SARS-CoV-2 infection have been the subject of publication in this Special Issue. Real-time PCR-based assays performed on respiratory secretions for the detection of viral RNA are currently considered the gold-standard method for SARS-CoV-2 diagnosis. Moreover, additional and more advanced molecular methods, such as droplet-digital PCR (ddPCR), clustered regularly interspaced short palindromic repeats (CRISPR), and next-generation sequencing (NGS), are currently under development to detect SARS-CoV-2 RNA in clinical specimens [8,15]. However, the high rate of single and multiple mismatches found in the target regions of molecular assays used worldwide for SARS-CoV-2 diagnosis reinforces the need to optimize and constantly update these assays according to SARS-CoV-2 genetic evolution and the future emergence of novel variants [16]. In this context, the omicron variant has been highly contagious worldwide, although the severity of the disease has been milder, with a less lethal course for patients. Recent diagnostic strategies have been adopted to either detect viral antigens, i.e., antigen-based immunoassays or human anti-SARS-CoV-2 antibodies, i.e., antibody-based immunoassays, in nasal or oropharyngeal swabs, as well as in blood or saliva samples, even using SARS-CoV-2 serologic methods [8,17]. An aspect that has been uncovered only partially, but may soon be the subject of in-depth studies, concerns the dosage of mucosal secretory IgA, especially at the ocular level. The role of mucosal IgA in counteracting SARS-CoV-2 infection, particularly at this site of virus entry, appears to be promising [18]. Ultimately, innovative tests such as MqSOFA and NEWS-2 were applied to assess intra-hospital mortality (IHM) and 30-day COVID-19 mortality. MqSOFA, although not able to predict treatment, is easier to use than NEWS-2, especially in the emergency setting and when appropriate [5,6].

There are still many uncertainties regarding knowledge of the virus and disease: are asymptomatic people actually infectious and with what charge? Can swabs alone be considered effective? How long will the vaccine be able to protect us? Why are people infected with SARS-CoV-2 not protected against a second infection? Will the immune shield acquired by the most vaccinated countries be sufficient to protect us from possible variations generated by poorly vaccinated countries? Nor do we know whether individuals who have developed severe symptoms of COVID-19 develop more neutralizing antibodies and thus greater protection against reinfection. Precisely in the case of coronavirus, protection may be short-lived, as several authors have suggested. However, it is also true that high levels of antibodies to pre-existing coronaviruses are associated with mild disease, suggesting that their measurement could be useful in predicting disease severity [3]. Epigenetics can help us answer some questions. Epigenetic disturbances in both the host and viruses are a matter of great interest in revealing the disparities in mortality and pathology of COVID-19. In fact, several targets of epigenetic modification in the virus, as well as the host, have been identified, including m6A, ACE2 modifications, etc., which have been linked to various conditions in infected hosts and their pathology [3,19]. Finally, considering the emergence of new variants, especially from countries where there has not been much stratification of vaccination, how will the situation evolve in general? From the literature data, it appears that breakthrough infections can significantly enhance α-S- and neutralizing antibody responses, indicating a possible benefit from booster vaccinations [9,10,20]. It is not excluded that many of these questions may be answered in the second edition (2023) of this Special Issue.

## References

[1] Contini C., Di Nuzzo M., Barp N., Bonazza A., De Giorgio R., Tognon M., Rubino S. (2020). The novel zoonotic COVID-19 pandemic: An expected global health concern. J. Infect Dev. Ctries..

[2] https://www.who.int/emergencies/diseases/novel-coronavirus-2019.

[3] Cugno M., Meroni P.L., Consonni D., Griffini S., Grovetti E., Novembrino C., Torri A., Griffante G., Gariglio M., Varani L. (2022). Effects of Antibody Responses to Pre-Existing Coronaviruses on Disease Severity and Complement Activation in COVID-19 Patients. Microorganisms.

[4] Rabaan A.A., Aljeldah M., Shammari B.R.A., Alsubki R.A., Alotaibi J., Alhashem Y.N., Alali N.A., Sulaiman T., Alsalem Z., Bajunaid H.A. (2023). Epigenetic Targets and Pathways Linked to SARS-CoV-2 Infection and Pathology. Microorganisms.

[5] Guarino M., Perna B., Remelli F., Cuoghi F., Cesaro A.E., Spampinato M.D., Maritati M., Contini C., De Giorgio R. (2022). A New Early Predictor of Fatal Outcome for COVID-19 in an Italian Emergency Department: The Modified Quick-SOFA. Microorganisms.

[6] Fernandez Chávez A.C., Aranaz-Andrés J.M., Roncal-Redin M., Roldán Moll F., Estévez Rueda M.J., Alva García P., Aranda García Y., San Jose-Saras D., on behalf Health Outcomes Research Group of the Instituto Ramón y Cajal de Investigación Sanitaria (IRYCIS) (2023). Impact of the COVID-19 Pandemic on Inappropriate Use of the Emergency Department. Microorganisms.

[7] Tokic D., Mikacic M., Kumric M., Ticinovic Kurir T., Rancic-Vidic I., Martinovic D., Bukic J., Vrdoljak J., Lizatovic I.K., Stipic S.S. (2022). Association between Brain Injury Markers and Testosterone in Critically-Ill COVID-19 Male Patients. Microorganisms.

[8] Rotondo J.C., Martini F., Maritati M., Caselli E., Gallenga C.E., Guarino M., De Giorgio R., Mazziotta C., Tramarin M.L., Badiale G. (2022). Advanced Molecular and Immunological Diagnostic Methods to Detect SARS-CoV-2 Infection. Microorganisms.

[9] Peter J.K., Wegner F., Gsponer S., Helfenstein F., Roloff T., Tarnutzer R., Grosheintz K., Back M., Schaubhut C., Wagner S. (2022). SARS-CoV-2 Vaccine Alpha and Delta Variant Breakthrough Infections Are Rare and Mild but Can Happen Relatively Early after Vaccination. Microorganisms.

[10] Streibl B.I., Lahne H., Grahl A., Agsten P., Bichler M., Büchl C., Damzog M., Eberle U., Gärtner S., Hobmaier B. (2022). Epidemiological and Serological Analysis of a SARS-CoV-2 Outbreak in a Nursing Home: Impact of SARS-CoV-2 Vaccination and Enhanced Neutralizing Immunity Following Breakthrough Infection. Microorganisms.

[11] Svyatchenko V.A., Ternovoi V.A., Lutkovskiy R.Y., Protopopova E.V., Gudymo A.S., Danilchenko N.V., Susloparov I.M., Kolosova N.P., Ryzhikov A.B., Taranov O.S. (2023). Human Adenovirus and Influenza A Virus Exacerbate SARS-CoV-2 Infection in Animal Models. Microorganisms.

[12] Guarino M., Perna B., Cuoghi F., Spampinato M.D., Cesaro A.E., Manza F., Pretula A., Grilli A., Maritati M., Caio G. (2022). Role of Intracellular Pulmonary Pathogens during SARS-CoV-2 Infection in the First Pandemic Wave of COVID-19: Clinical and Prognostic Significance in a Case Series of 1200 Patients. Microorganisms.

[13] Wang J., Mao J., Chen G., Huang Y., Zhou J., Gao C., Jin D., Zhang C., Wen J., Sun J. (2021). Evaluation on blood coagulation and C-reactive protein level among children with mycoplasma pneumoniae pneumonia by different chest imaging findings. Medicine.

[14] Vainas T., Kurvers H.A., Mess W.H., de Graaf R., Ezzahiri R., Tordoir J.H., Schurink G.W., Bruggeman C.A., Kitslaar P.J. (2002). Chlamydia pneumoniae serology is associated with thrombosis-related but not with plaque-related microembolization during carotid endarterectomy. Stroke.

[15] Rotondo J.C., Martini F., Maritati M., Mazziotta C., Di Mauro G., Lanzillotti C., Barp N., Gallerani A., Tognon M., Contini C. (2021). SARS-CoV-2 Infection: New Molecular, Phylogenetic, and Pathogenetic Insights. Efficacy of Current Vaccines and the Potential Risk of Variants. Viruses.

[16] Alkhatib M., Carioti L., D’Anna S., Ceccherini-Silberstein F., Svicher V., Salpini R. (2022). SARS-CoV-2 Mutations and Variants May Muddle the Sensitivity of COVID-19 Diagnostic Assays. Microorganisms.

[17] Jeske R., Merle U., Müller B., Waterboer T., Butt J. (2022). Performance of Dried Blood Spot Samples in SARS-CoV-2 Serolomics. Microorganisms.

[18] Caselli E., Soffritti I., Lamberti G., D’Accolti M., Franco F., Demaria D., Contoli M., Passaro A., Contini C., Perri P. (2020). Anti-SARS-CoV-2 IgA Response in Tears of COVID-19 Patients. Biology.

[19] Behura A., Naik L., Patel S., Das M., Kumar A., Mishra A., Nayak D.K., Manna D., Mishra A., Dhiman R. (2023). Involvement of epigenetics in affecting host immunity during SARS-CoV-2 infection. Biochim. Biophys. Acta (BBA)-Mol. Basis Dis..

[20] Al-Hatamleh M.A., Abusalah M.A., Hatmal M.M., Alshaer W., Ahmad S., Mohd-Zahid M.H., Rahman E.N.S.E., Yean C.Y., Alias I.Z., Uskoković V. (2023). Understanding the challenges to COVID-19 vaccines and treatment options, herd immunity and probability of reinfection. J. Taibah Univ. Med Sci..

